# Processing and Utilization Technology of Root and Tuber Food

**DOI:** 10.3390/foods13132082

**Published:** 2024-07-01

**Authors:** Fankui Zeng, Huachun Guo, Gang Liu

**Affiliations:** 1 Research Center for Natural Medicine and Chemical Metrology, Lanzhou Institute of Chemical Physics, Chinese Academy of Sciences, Lanzhou 730000, China; gangliu@licp.cas.cn; 2 College of Agronomy and Biotechnology, Yunnan Agricultural University, Kunming 650000, China; ynghc@126.com

## 1. Introduction

Roots and tubers make a great contribution to major staple foods and provide good sources of dietary carbohydrates for the nutrition supply and energy recharge of human [1]. These crops include cocoyam (*Colocasia* spp. and *Xanthosoma* spp.), potato (*Solanum tuberosum*), sweet potato (*Ipomea batatas*), yams (*Dioscorea* spp.), and cassava (*Manihot esculenta*), which are the most cultivated species after cereals and legumes [2]. As for root and tuber crops, they are constituted by 70–80% water, 16–24% starch, and less than 4% proteins and lipids [3]. The root and tuber crops showed a steady increase in production and plant areas. According to FAO statistics, in 2022, the world harvested areas of cassava, potato, sweet potato, and yam were 32.04, 17.78, 7.24, and 10.4 million hectares, respectively, and their world yields were 3304.1, 3747.78, 864.1, and 882.57 hundred million kilograms, respectively. Due to the importance of root and tuber crops in food, nutrition, and cash income, they are even listed in the RTB (Root, Tuber, and Banana) breeding program together as a contributing source of food for their quality characteristics and potential benefits [4]. Thus, new high-yielding and disease-resistant varieties will be developed in the future [5,6].

In recent years, a large number of studies have focused on the isolation, modification, physicochemical properties, and applications of root and tuber crops [7,8]. The excellent properties of starch in root and tuber crops, including environmental benignity, easy fabrication, relative abundance, non-toxicity, and biodegradability, have received more attention. However, as the native starch has a defect of poor thermo-mechanical properties and higher water absorptivity, more researchers are taking time to modify and apply starches from different crops [9,10,11]. For instance, the starch derived from *Pueraria lobate* is limited due to its inherent properties, so the kudzu starch is not suitable for the food industry and need to be further processed [12]. Through a series of modification methods, the rheological, functional, and processing characteristics can be used to improve physical and chemical, textural, and edible properties. Therefore, the deep processing of various modified starch and the related products obtained great market potential [13]. In addition, the flour from root and tuber crops also has an essential application. With the worldwide challenges of food security, nutrition, and sustainable agriculture, composite flour development and utilization are hell-bent on winning, and it has emerged as a promising strategy [14]. The composite flours represent mixtures of different flours from potato, sweet potato, cassava, and yam and flours or cereals that have enriched proteins, which can be used to meet specific functional properties and nutritional requirements [15]. This technology concerning composite flour was proposed in 1964, which largely reduced the reliance on wheat flour [16]. Additionally, it is a way of fostering agro-industries, stimulating more viable crops to be cultivated [17]. In a word, composite flour can provide human beings with more nutritional components, such as minerals, vitamins, dietary fibers, carbohydrates, and proteins, compared with single cereal flour. Jenfa et al. demonstrated that substituting sorghum with orange-fleshed sweet potato flour significantly elevated the vitamin A content in sorghum-based food products [18]. As mentioned above, each kind of tuber or root crop possesses diverse nutrition functions and application potential [19,20], so studying the processing and utilization technology of root and tuber food will offer valuable information about new application technologies and reveal deep mechanisms of nutrition exploration.

## 2. An Overview of the Published Articles

Ekumah et al. (Contribution 1) discussed the influence of ethanol concentration and exposure time on kudzu starch gel production and determined the starch gel’s mechanical, rheological, and digestibility properties. This treatment has the benefit of optimizing starch-based products and is also helpful for the implications in the confectioneries, desserts, beverages, and pharmaceuticals fields. In detail, the properties of robust starch gels using ethanol exposure were assessed by textural, rheological, structural, and in vitro digestion. The results showed that the starch-based gel’s strength was improved significantly by 187%, and the thermal transitions were elevated with the increase in ethanol. The ethanol exposure resulted in a reduction in syneresis in size and color, and the elasticity of gels was obviously improved. The X-ray result indicated that the B- and V-type patterns were more stable with an increase in relative crystallinity. In conclusion, the exposure to ethanol is essential for improving the mechanical characteristics of kudzu starch gels; meanwhile, the higher content of resistant starch fractions were well preserved, which proved very well the ethanol’s transformative impact on the diverse properties of kudzu starch gels and provided an important theoretical basis for the application of starch-based products.

The article by Li et al. (Contribution 2) makes a comprehensive assessment of the functional characteristics of wheat–cassava composite flour. Cassava flour has the advantage of low retrogradation, high water-binding ability, and high fiber and mineral content. It has been used in a large amount of processed foods, e.g., cakes, biscuits, bread, and noodles, and so on. After replacing part of wheat flour with cassava flour, the final products offer consumers more carbohydrates and low-lipid foods, leading to a lower glycemic response. In this work, the levels of 0% (control), 10%, 20%, 30%, 40%, and 50% wheat flour were substituted by cassava flour, the functional properties of which showed that the moisture, protein, fat, and b* value of composite flour declined with the increase of cassava flour, but the crude fiber, ash, starch, L*, a* values, iodine blue value (IBV), and swelling power of that flour increased instead. This composite flour resulted in a decline in elasticity and cohesiveness when the cassava content was from 10% to 30%. From this research, we can obtain important information on selecting the appropriate substitution levels of cassava for different products.

The third article was contributed by Liu et al. from the University of Shanghai for Science and Technology (Contribution 3). Fried foods are always popular all over the world and also are well liked by most people. However, the high level of fat and calorie intake raises concerns about hypertension, cardiovascular disease, diabetes, and cancer risk. Also, the traditional frying led to a high content of acrylamide due to the high temperature of more than 200 °C. Therefore, high-quality fried foods with lower fat and acrylamide are strongly needed in the current situation. The preliminary treatment by pulsed electric fields and blanching was found to significantly affect the final sensory properties of fried sweet potato chips. Importantly, the pulsed electric fields with 0.2 s duration and 1 Kv/cm intensity in combination with blanching pretreatment at 85 °C significantly reduced the acrylamide level by approximately 46.1%. And the final fried potato chips displayed more smoothness and flatness. Regarding the single blanching pretreatment, it was useful for reducing moisture ratio, oil intake, and the change in color. The hardness certainly increased. In conclusion, these combined treatments are feasible for the industrial production of root vegetables that are fit to fry, by which the high quality and low cost of products can be easily controlled and achieved in the future.

The fourth article (Contribution 4) also focused on the application of composite flour, as in the second article. Its main content concerns the application of composite flour derived from Indonesian local tubers into a kind of pancake. Pancakes serve as a kind of fast and easy food snack that is made based on wheat flour. There is a protein called gluten in wheat flour, which is helpful for the cohesiveness, viscosity, and elasticity of dough. However, this specific protein is disadvantageous to the human population that suffers from celiac disease. The presence of gluten protein can cause metabolic disorders and the occurrence of disease. Therefore, it needs to replace the wheat flour with other flours. In the present work, a kind of free-gluten pancake using alternative flours from local cassava, arrowroot, and suweg was produced. The obtained data showed that the ratio of three different flours had a significant effect on the functional properties such as pasting temperature, peak viscosity, hold viscosity, breakdown viscosity, setback, L*, a*, hue, whiteness, ∆E, as well as the solubility of composite flour. Moreover, the ration also affects the texture, color, and flavor characteristics of free-gluten pancakes. Based on the provided results, especially the evaluation of panelists, the pancakes made with alternative tuber flours were acceptable with good traits, which are basically similar with to those made from wheat flour. It is acknowledged that the composite flours are required in future food industries.

The fifth article published in this Special issue is a review by Xu et al. (Contribution 5) on functional foods based on potato. Potatoes are planted worldwide and make great contributions to food security and the food industry. This article reviewed the prominent components, including starch, protein, phytochemicals, and minerals, in detail, the application of starch and protein, the functions of different ingredients, and looked forward to the future development of potato research. It also proposed several main tasks. In the future, the starch-based products for special groups, the fiber-rich products for obese people, the bio-friendly films of starch, and the bioactive proteins will be new commercial products on the market. After reading this review, the whole story, from the knowledge of diverse nutritional ingredients to the application of starch and protein, can be clearly understood. Importantly, this review provided valuable information and pointed out a definite orientation for a series of essential studies.

## 3. Conclusions

The root and tuber crops belong to staple foods and are cultivated over a wide variety of soils. The flours and starches derived from root and tuber crops have elicited great use in the food and non-food industries. The diversity in these crop genotypes accounts for differences in end-product properties and would further require the characterization of varieties for the suitability of culinary and processing methods. However, the application of starches and flours in food processing is determined by their inherent properties, including composition, physicochemical, and functional properties. The texts described above are all related to the functional properties of starch-based products or new products using composite flours. Among them, one article discussed the effect of ethanol exposure on the kudzu starch gels by analyzing their mechanical characteristics and digestibility and proved their potential application in various fields in industries. Moreover, two articles both verified the functional properties of different composite flours and their effective application in processed products. Another paper is about the effect of combined treatment on the fried sweet potato chips. All of these advanced technologies bring us new products that are beneficial and healthy for human populations.

Thus, the published articles in this Special Issue should not only be an investigation result but also an orientation and trend for future research on root and tuber crops. With these focuses on processed products such as potato chips, sweet potato chips, French fries, and dehydrated potatoes, the need for more scientific studies towards the development of novel foods related to root or tuber crops is necessary. Therefore, there are some directions that need further effort: (1) optimize the distribution of raw materials in order to enrich the resources for food products; (2) expand the raw material production by variety selection and breeding; (3) develop diversified staple food products according to various consumer demands by strengthening the technologies; (4) guide consumers to consume novel products with nutritional functions by positive publicity and popularization; and (5) increase the investment in scientific research by linking up processing and marketing.

Finally, I would like to highlight the particularity that was shown in all five articles. Except for a review, the other four texts are focused on the functional properties of starch-based products and composite flours form different root or tuber crops, which were less proposed in the previous studies. Thus, it was noted that this Special Issue is of importance and worth reading for readers to know about the research emphasis and development progress in the field of root and tuber crops, which also provide an information platform for researchers to view academic dynamics in this orientation.
